# Relationship between electrically evoked compound action potential thresholds and behavioral T-levels in implanted children with cochlear nerve deficiency

**DOI:** 10.1038/s41598-023-31411-3

**Published:** 2023-03-15

**Authors:** Xiuhua Chao, Ruijie Wang, Jianfen Luo, Haibo Wang, Zhaomin Fan, Lei Xu

**Affiliations:** Department of Otolaryngology-Head and Neck Surgery, Shandong Provincial ENT Hospital,, Shandong University, Jinan, 250022 People’s Republic of China

**Keywords:** Paediatric research, Peripheral nervous system

## Abstract

It is challenging to program children with cochlear nerve deficiency (CND) due to limited auditory and speech abilities or concurrent neurological deficits. Electrically evoked compound action potential (ECAP) thresholds have been widely used by many audiologists to help cochlear implant programming for children who cannot cooperate with behavioral testing. However, the relationship between ECAP thresholds and behavioral levels of cochlear nerve in children with CND remains unclear. This study aimed to investigate how well ECAP thresholds are related to behavioral thresholds in the MAP for children with CND. This study included 29 children with CND who underwent cochlear implantation. For each participant, ECAP thresholds and behavioral T-levels were measured at three electrode locations across the electrode array post-activation. The relationship between ECAP thresholds and behavioral T-levels was analyzed using Pearson’s correlation coefficient. The results showed that ECAP thresholds were significantly correlated with behavioral T-levels at the basal, middle, and apical electrodes. ECAP thresholds were equal to or higher than the behavioral T-levels for all tested electrodes, and fell within MAP’s dynamic range for approximately 90% of the tested electrodes. Moreover, the contour of the ECAP thresholds was similar to the contour of T-levels across electrodes for most participants. ECAP thresholds can help audiologists select stimulation levels more efficiently for children with CND who cannot provide sufficient behavioral response.

## Introduction

Cochlear nerve deficiency (CND) refers to an absent or small cochlear nerve. CND is diagnosed based on the magnetic resonance imaging (MRI) results. It is diagnosed as cochlear nerve aplasia if the cochlear nerve could not be identified on any plane of the MRI, and cochlear nerve hypoplasia is diagnosed if the diameter of the cochlear nerve is smaller than that of the adjacent facial nerve^1,2^. Children with CND often have severe-to-profound sensorineural hearing loss (SNHL), and cochlear implantation is the main treatment for them. However, the outcomes of cochlear implants (CI) in children with CND were poorer than those who have a normal-sized cochlear nerve, and varied greatly among individual patients^3–5^. It has been reported that approximately half of the children with CND can acquire some spoken language after long-term rehabilitation, but there still a few children only had the sound detection ability improved^6^.

Despite many previous studies have reported poor outcomes of cochlear implantation, there remains limited data regarding the programming of speech processors in children with CND. A well-fitted speech processor is critical to the postoperative hearing outcomes. Previous studies have shown that the responsiveness of the cochlear nerve to electrical stimulation was reduced in children with CND than in other SNHL children with normal-sized cochlear nerves^7,8^. This indicated that the programming parameters in children with CND might be different from other SNHL children. Furthermore, substantial variations in the functional status of the cochlear nerves among CND children have also been reported^7,9^. Therefore, specific parameters might be required to generate a high-quality hearing effect for a particular child with CND. Typically, the programming of a speech processor is based on behavioral responses to set appropriate stimulation levels. However, obtaining reliable behavioral responses from children with CND is challenging, since more than half of these children have concurrent neurological deficits^10^. In the clinic, cochlear implant programming for children with CND remains a stiff issue.

Electrically evoked compound action potential (ECAP) thresholds have been widely used by many audiologists to help program behavioral threshold (BT) and maximum comfortable hearing levels (C-level/M-level) in speech processor MAPs in young children^11–13^. Although there is some controversy on how well ECAP thresholds predict MAP’s T or C-levels, multiple studies showed significant correlations between the ECAP thresholds and behavioral levels in children with normal-sized cochlear nerves^14–16^. These results allow audiologists to select stimulation levels more efficiently. To date, whether ECAP responses could be used to assist programming a cochlear implant processor in children with CND has not been reported. Whether the relationship between ECAP thresholds and behavioral levels of cochlear nerve in children with CND is similar to other SNHL children remains unclear. Thus, this study aimed to investigate how well ECAP thresholds are related to behavioral T-levels in children with CND.

## Materials and methods

This cohort study was approved by the ethics committee of Shandong Provincial ENT Hospital (No. XYK20170906). Informed consent was obtained from all participants’ legal guardians prior to participation. All experiments were performed in accordance with relevant guidelines and regulations, and the Declaration of Helsinki. This study followed all requirements listed in the strobe statement.

### Participants

This study included 29 children diagnosed with CND who underwent cochlear implantation in our center (CND1–CND29). Participants were recruited according to the following criteria: (1) diagnosed as bilateral SNHL and CND before implantation; (2) implanted with a Cochlear^®^ Nucleus device (Cochlear Ltd., Sydney, Australia); (3) had been regularly programmed in our center for more than 1 year; (4) could provide reliable behavioral responses; (5) ECAPs could be recorded in some of their electrodes. The exclusion criteria included: (1) children who had the electrode array been partially implanted; (2) children who had CND combined with cochlear malformations (such as Common Cavity, Cochlear Hypoplasia or Incomplete Partitions); (3) children with no ECAP responses recorded from any of the electrodes. All participants were implanted with a contour electrode array, either 24RE[CA] or CI512, in the test ear. The participants’ age at implantation ranged from 1.0 to 9.6 years (mean: 3.1 years; standard deviation (SD): 2.7 years). All participants wore their sound processor more than eight hours every day, and had a minimum of 1 year of listening experience with CI before participating. The tested ages ranged from 2.6 to 12.2 years (mean: 6.9 years; SD: 2.5 years). The anatomical status of the cochlear nerve and cochlea were assessed based on results of MRI and high-resolution computed tomography (HRCT) scans following previously described protocols^3^. For all participants, bilateral cochlear nerves were absent on the MRI scans, and bilateral cochlear formations were normal on HRCT scans. Detailed demographic information of these participants is listed in Table 1.

**Table 1:** Subject biographical, cochlear implant and MAP’s parameters information. *AAI* age at implantation, *AAT* age at testing, *pps* pulse per second, *ECAPs* electrically evoked compound action potentials.

Subject number	Gender	Ear tested	AAI (years)	AAT (years)	Electrode array	Processor	active electrodes	Rate (pps)	Pulse Width (μs)	Tested electrodes	Electrodes with ECAPs
CND1	F	R	3.4	6.8	24RE (CA)	Freedom	All	720	37	1, 4, 8	1–8
CND2	M	R	6.1	11.4	24RE (CA)	Freedom	All	500	50	3, 12, 21	1–22
CND3	M	L	6.0	7.8	24RE (CA)	Freedom	All	500	50	1, 3, 7	1–7
CND4	M	L	6.5	8.5	24RE (CA)	CP802	All	500	50	3, 12, 21	1–22
CND5	F	L	3.9	6.9	24RE (CA)	Freedom	All	500	50	6, 15, 21	6–22
CND6	M	L	1.9	3.9	24RE (CA)	CP802	All	500	50	3, 9, 15	1–15
CND7	M	L	2.1	6.1	24RE (CA)	Freedom	All	500	50	3, 12, 21	1–22
CND8	F	L	6.0	10.2	24RE (CA)	Freedom	All	500	50	3, 12, 21	1–22
CND9	F	L	1.0	2.6	24RE (CA)	Freedom	All	500	50	3, 12, 21	1–22
CND10	F	R	2.4	7.2	24RE (CA)	CP910	All	500	50	3, 12, 21	1–22
CND11	M	R	6.6	9.0	24RE (CA)	Freedom	All	500	50	1, 6, 10	1–10
CND12	M	L	9.6	12.2	24RE (CA)	Freedom	All	500	50	3, 12, 21	1–22
CND13	M	L	1.3	3.9	24RE (CA)	Freedom	All	720	37	3, 12, 21	1–22
CND14	M	R	1.3	3.9	24RE (CA)	Freedom	All	720	37	3, 12, 21	1–22
CND15	M	L	3.5	4.7	24RE (CA)	CP802	All	500	50	3, 12, 21	1–22
CND16	F	L	1.1	3.3	24RE (CA)	Freedom	All	500	75	2, 10, 20	1–21
CND17	F	L	4.4	8.9	24RE (CA)	Freedom	All	500	50	1, 3, 5	1–5
CND18	F	L	2.6	3.7	CI512	CP910	1–15	500	50	2, 6, 15	1–15
CND19	F	L	4.7	7.5	24RE (CA)	Freedom	All	500	50	1, 12, 21	1–22
CND20	M	L	6.0	8.1	24RE (CA)	Freedom	All	500	50	3, 12, 21	1–22
CND21	M	R	3.9	7.5	24RE (CA)	Freedom	1–15	500	75	1, 6, 11	1–11
CND22	M	L	3.2	7.7	CI512	CP910	All	500	75	1, 6, 12	1–12
CND23	M	R	3.9	7.7	CI512	CP910	All	500	75	1, 5, 10	1–10
CND24	M	L	3.6	9.1	24RE (CA)	Freedom	All	720	37	3, 12, 21	1–22
CND25	F	L	3.0	4.7	24RE (CA)	Freedom	All	500	50	3, 12, 21	1–22
CND26	F	L	1.3	4.1	24RE (CA)	Freedom	All	500	50	3, 12, 21	1–22
CND27	F	R	2.9	8.9	24RE (CA)	Freedom	All	500	50	3, 6, 9	1–9
CND28	F	R	4.6	8.3	24RE (CA)	Freedom	All	720	37	3, 12, 20	1–20
CND29	F	R	1.9	6.1	24RE (CA)	Freedom	All	500	50	1, 6, 11	1–11

### ECAP measurements

**Figure 1:**
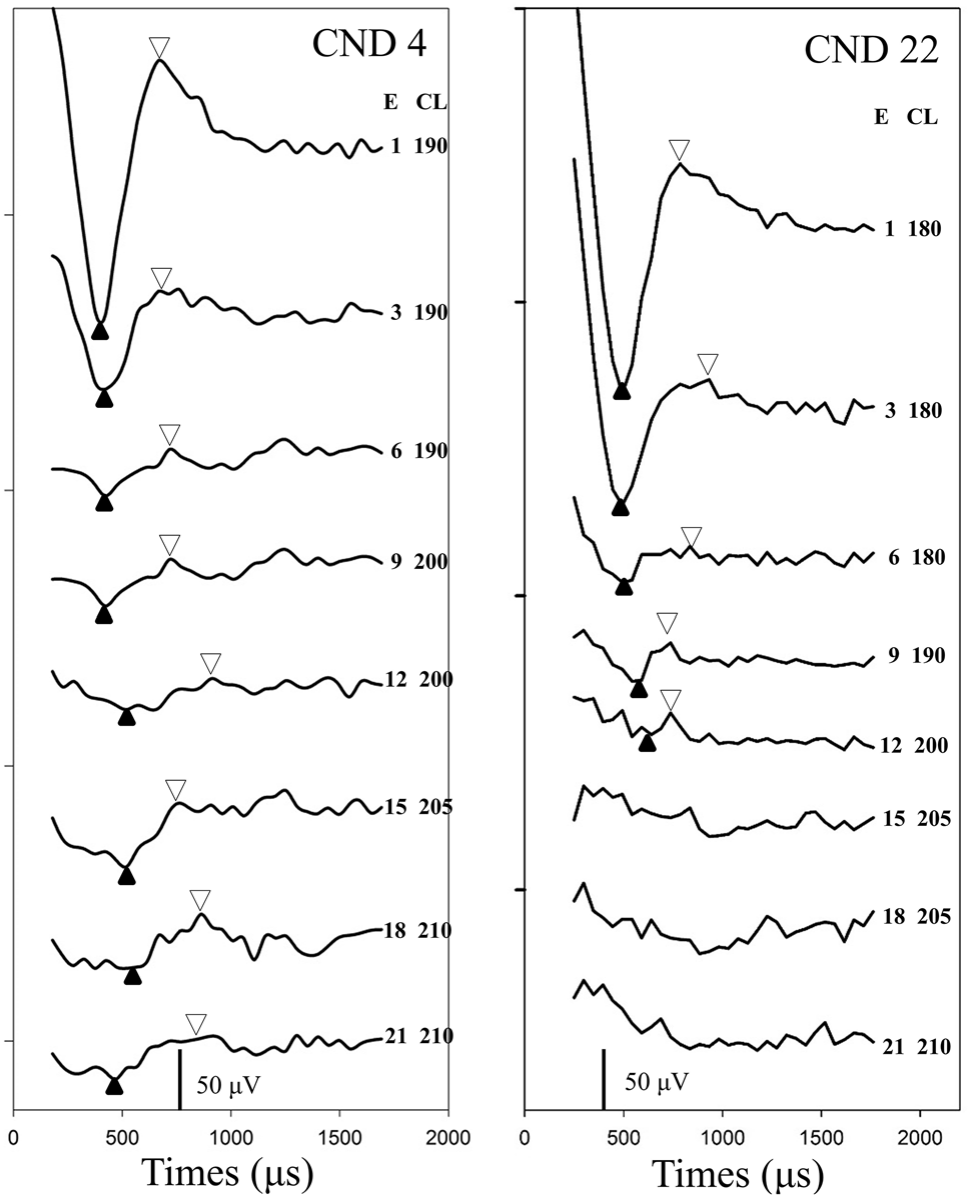
Traces recorded at some electrodes in CND4 and CND22. Subject number, the electrode location and stimulation level (current level, CL) used to evoke these traces are displayed on each panel. The pulse width used in CND4 and CND22 was 50 μs and 75 μs, respectively. The upward and downward triangle indicate the trough and peak of the electrically evoked compound action potential identified for the trace, respectively. *E* electrode location, *CL* current level.

ECAPs were measured using the advanced neural response telemetry function provided by the Custom Sound EP (v. 4.3) software (Cochlear Ltd.). For each participant, the maximum acceptable levels which would not evoke uncomfortable responses (such as fear, blinking, twitching and crying) were tested for each electrode before the ECAP recording. The two pulses forward masking method was used to record ECAP waveforms in this study. Parameters used to record ECAP were following previously described protocol^7^. The stimulus was a single cathodic-leading, biphasic, charge-balanced pulse. The probe rate was 15 Hz, the pulse width varied across individuals from 37 to 75 μs/phase, and the inter-phase-gap was 7 μs. The recording electrode was two or three electrodes away in the basal direction from the stimulating electrode, with a sampling delay of 98–142 μs. These parameters were adjusted for each participant to obtain typical ECAP morphologies. First, we attempted to record ECAP waveforms from each electrode along the electrode array. Figure 1 demonstrated traces recorded at some electrode locations in two children. For CND4, ECAPs could be recorded from all electrodes. For CND22, ECAP could only be recorded from electrodes 1 to 12.

**Figure 2:**
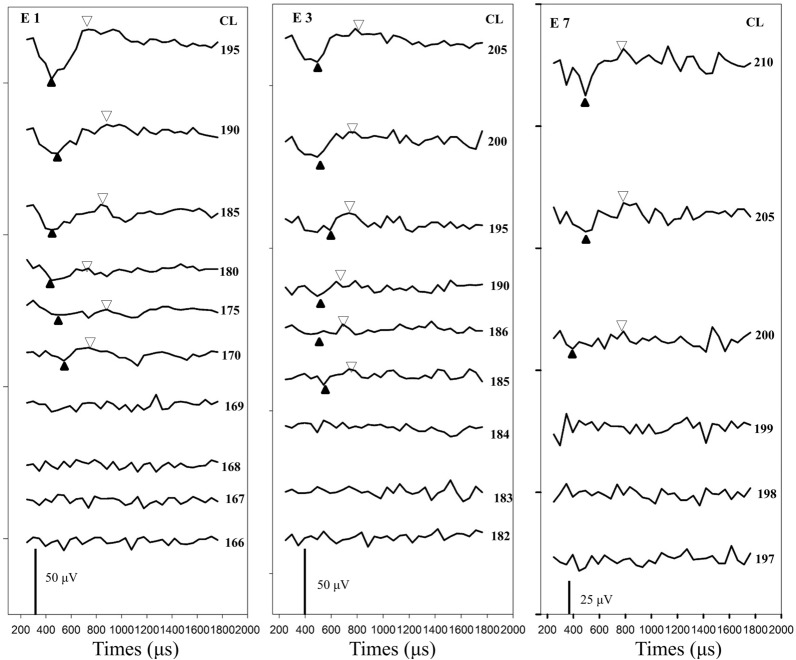
This figure shows waveforms of ECAP responses recorded for three stimulating electrodes (1, 3 and 7) in CND17. Each panel shows results measured in one electrode. Stimulation levels used to evoke each trace are labeled at the right side of the panel. The pulse width used in each electrode was 50 μs.

Then, the ECAP input/output function (I/O function) was measured at three electrode locations where ECAP waveforms could be recorded. For Cochlear Nucleus devices, there are 22 electrodes alone the electrode array with electrode 1 placed near the basal of the cochlea and electrode 22 placed near the apical of the cochlea. For participants whose ECAPs could be recorded at all electrode locations, electrodes 3, 12, and 21 were selected. For participants whose ECAPs could only be recorded at some electrode locations, the selected electrodes were extended to the most apical electrode location with a measurable ECAP, and testing electrodes were relatively equally separated. These selected electrodes were considered the basal, middle, and apical electrodes in this study. For the ECAP I/O function, the probe level started at the maximum acceptable level and decreased in steps of five current levels (CL) until no response could be visually identified, and subsequently increased in steps of one CL until continuous ECAPs could be measured using this small step size. Figure 2 shows the ECAP I/O function waveforms tested at three electrode locations for CND17. All ECAP thresholds were determined based on a mutual agreement between two audiologists who reviewed the data independently. For each participant, it took approximately two hours to collect all the ECAP thresholds data.

### Cochlear implant programming and behavioral T-levels testing

Regular programming was performed postoperatively for each participant. The speech processor used was Freedom, Nucleus 5 or Nucleus 6 (Cochlear, Ltd.). The default pulse width and stimulation rate used in the Freedom or Nucleus speech processor were 25 μs/phase and 900 pulse per second (pps), respectively. The pulse widths used in this study were 37–75 μs/phase, and the stimulation rates used were 500 or 720 pps. The strategy used was the advanced combination encoder, and the stimulation mode used was monopolar 1 plus 2 in all participants’ speech processors. T- and C-levels were set using approaches based on age and capacity to provide behavioral responses among individual participants, following previously described protocols^11,13^. T-levels were set such that the child responded confidently 100% of the time. C-levels were set to the maximum acceptable levels. For children who did not understand the concept of loudness of sound due to poor auditory-verbal skills, C-levels were set at the highest level such that the patient did not show any sign of discomfort. After programming, loud sounds were used to confirm children had comfortable listening experience. The C-levels would be mildly decreased as a whole if participants felt too loud or have non-sound stimulation. Moreover, the aided hearing threshold with CI and the detection of Ling’s six sounds were tested to verify whether children were well fitted. All behavioral and ECAP thresholds were tested during the same visit.

### Statistical analysis

In this study, the pulse width used for testing ECAP thresholds and behavioral T-levels was the same for each child, but it varied among the participants. Therefore, stimulation levels in the MAP and ECAP thresholds were converted to units of electrical charge per phase (nC). Pearson’s correlation coefficient was used to assess the correlation between ECAP thresholds and behavioral T-levels. In this study, the |r| ≥ 0.7 was considered that the two variables have strong correlation, 0.4 ≤ |r| < 0.7 was considered that the two variables have moderate correlation, |r| < 0.4 was considered that the two variables have weak or none correlation. The differences between the ECAP thresholds and behavioral T-levels across electrodes were assessed using repeated analysis of variance (ANOVA) test. Post-hoc comparisons were performed using Tukey’s pairwise test with Bonferroni correction. Statistical significance was set at p < 0.05.

## Results

### Relationship between ECAP thresholds and behavioral T-levels

**Figure 3:**
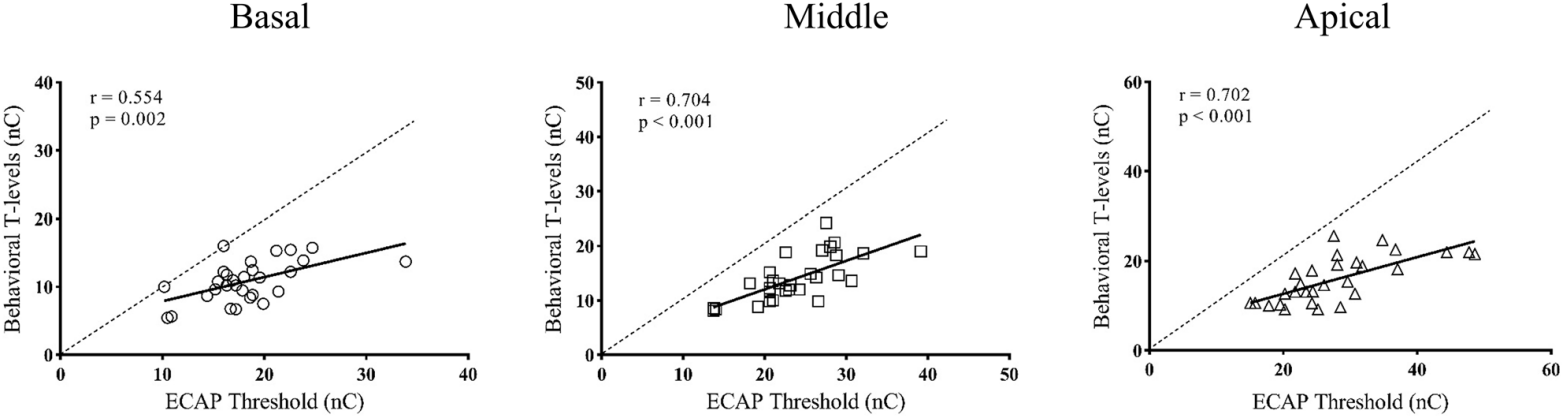
This figure shows a scatter plot of ECAP thresholds versus behavioral T-levels at the “Basal”, “Middle” and “Apical” electrode locations. The dotted line indicates that the ECAP threshold was equal to the behavioral T-level; the solid line represents the correlation between ECAP thresholds and behavioral T-levels.

ECAP response could be recorded at all activated electrodes in 14 participants; in the others, it could only be recorded at some of the electrodes. The percentage of measurable ECAP responses was 78.8%. As the electrode location moved from the basal to the apical relative to the cochlea, the potential to record the ECAP waveforms tended to decrease. Electrodes with ECAP response and tested electrodes for each participant are shown in Table 1. At the basal, middle, and apical electrode locations, ECAP thresholds were 18.27 (SD: 4.68; range: 10.50–33.87) nC, 23.80 (SD: 5.67; range: 13.70–39.14) nC, and 27.84 (SD: 8.68; range: 15.07–48.61) nC, respectively; and behavioral T-levels were 10.82 (SD: 2.97; range: 5.45–16.32) nC, 14.04 (SD: 4.22; range: 8.11–24.27) nC, and 15.89 (SD: 5.08; range: 9.15–25.63) nC, respectively. Repeated-measures ANOVA indicated that electrode location had a significant effect on ECAP thresholds (F_(2, 56)_ = 45.08, p < 0.01) and behavioral T-levels (F_(2, 56)_ = 37.32, p < 0.01). Post-hoc testing with Bonferroni correction showed that ECAP thresholds and behavioral T-levels tested at basal electrodes were significantly lower than those tested at middle (p < 0.01) and apical electrodes (p < 0.01); and ECAP thresholds and behavioral T-levels tested at middle electrodes significantly lower than those tested at apical electrodes (p < 0.01). Figure 3 shows the relationship between ECAP thresholds and behavioral T-levels at three electrode locations along the electrode array. There were significant correlations between ECAP thresholds and behavioral T-levels at the basal (r = 0.554, p = 0.002), middle (r = 0.704, p < 0.001), and apical (r = 0.702, p < 0.001) electrode locations.

### Daily MAPs of children with CND

**Figure 4:**
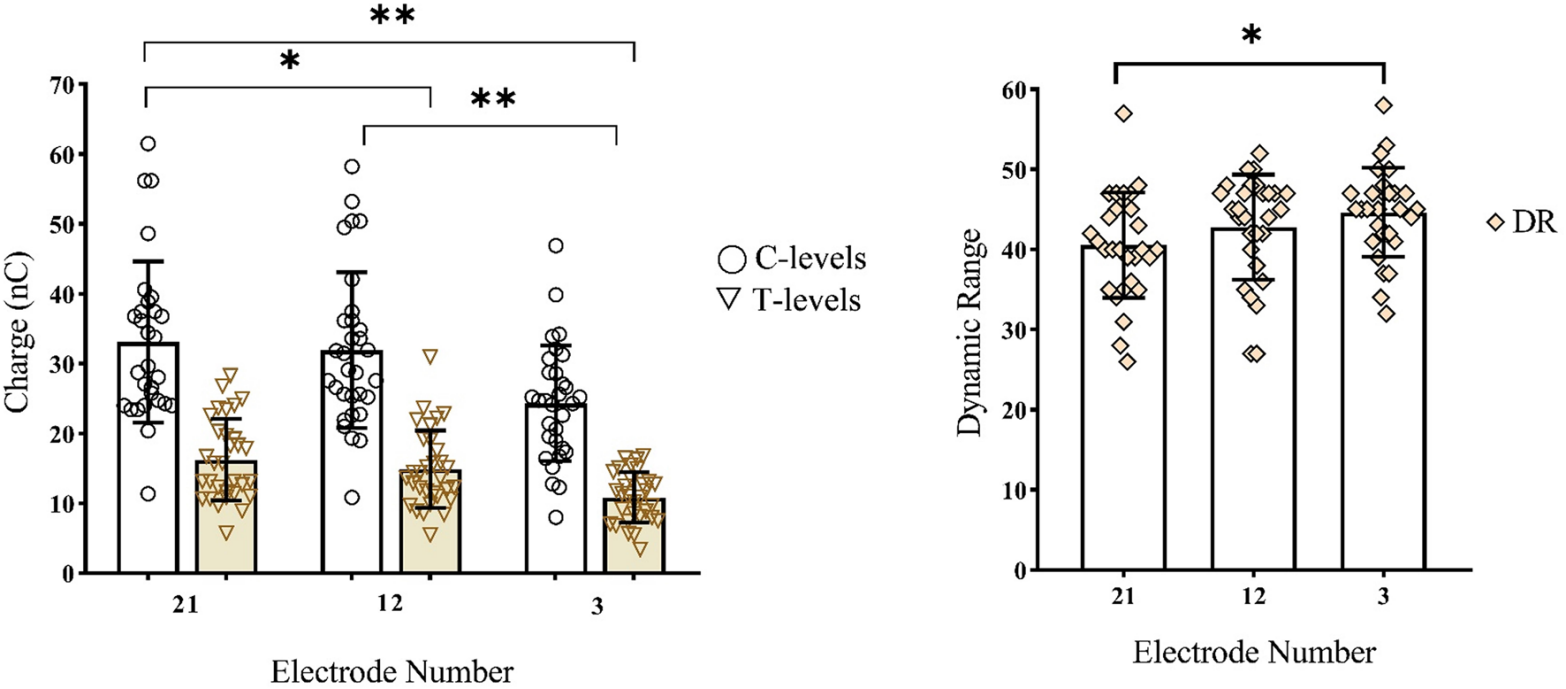
The left figure shows MAP’s C-levels and T-levels (in charge units), and the right figure shows MAP’s dynamic range (DR) at electrodes 3, 12, and 21. Round and triangle symbols in the left panel represent C-levels and T-levels for individual children with CND, respectively. The rhombuses in the right panel represent DRs for individual children with CND. The boxes represent mean scores. The error bars represent ± 1 SD. * represents p < 0.05; ** represents p < 0.01.

Part of the apical electrodes were deactivated in two participants (CND18 and CND21) due to facial stimulation. For all other participants, all electrodes were activated in their daily MAPs. MAP parameters in each participant’s speech processor are shown in Table 1. Figure 4 shows the means and SDs of T-levels, C-levels and dynamic ranges at electrodes 21, 12, and 3 in the daily MAPs of all participants except those two participants with only half electrodes activated. It is clear that from electrode 21 to electrode 3, T-levels and C-levels gradually decreased, while the dynamic ranges gradually increased. Repeated-measures ANOVA showed that electrode location had a significant influence on MAP’s T-levels (F_(2, 56)_ = 51.71, p < 0.01), C-levels (F_(2, 56)_ = 51.67, p < 0.01) and dynamic ranges (F_(2, 56)_ = 8.97, p < 0.01). Post-hoc testing with Bonferroni correction showed that MAPs’ C-levels and T-levels tested at electrode 21 were significantly higher than those tested at electrode 12 (p < 0.05) and electrode 3 (p < 0.01); and MAPs’ C-levels and T-levels tested at electrode 12 significantly higher than those tested at electrode 3 (p < 0.01). Additionally, significant differences in the DR measured between electrode 21 and electrode 3 were observed (p < 0.01). The aided hearing thresholds at 500 Hz, 1 K Hz, 2 K Hz and 4 K Hz with the MAP programmed at the testing time were between 20 dB HL to 35 dB HL for all participants.

### Position of ECAP thresholds in daily MAP’s dynamic range

**Figure 5:**
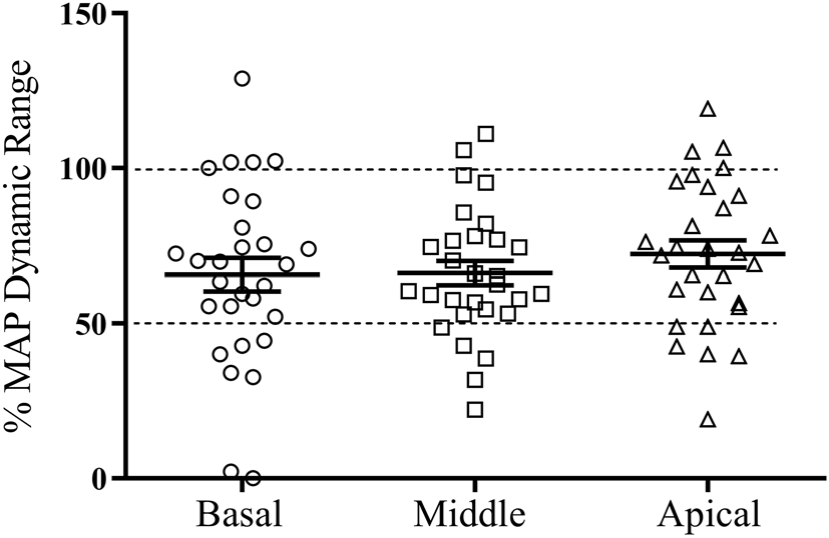
This figure shows a scatter plot of ECAP thresholds position in comparison to MAP’s dynamic range at the “Basal”, “Middle” and “Apical” electrode locations. The lower dotted line represents ECAP threshold fall at the middle of the MAP’s dynamic range, and the upper dotted line represents ECAP threshold is equal to the MAP’s comfortable level. The solid lines represent mean and standard error bar of the percentage of ECAP threshold to MAP’s dynamic range.

Figure 5 displays the position of ECAP thresholds in participants’ daily MAPs. MAPs’ T-levels were normalized to 0%, and C-levels were normalized to 100%. Overall, the ECAP thresholds were equal to or higher than the behavioral T-levels at all tested electrodes. Furthermore, the ECAP thresholds fell within MAPs dynamic range for approximately 90% (78/87) of the tested electrodes. However, ECAP threshold greatly scattered within the dynamic range, with approximately 70% of the tested electrodes falling at the upper panel of the MAP's dynamic range. The average position of ECAP thresholds within the dynamic range was approximately 65%, 66%, and 72% for basal, middle, and apical electrodes, respectively.

**Figure 6:**
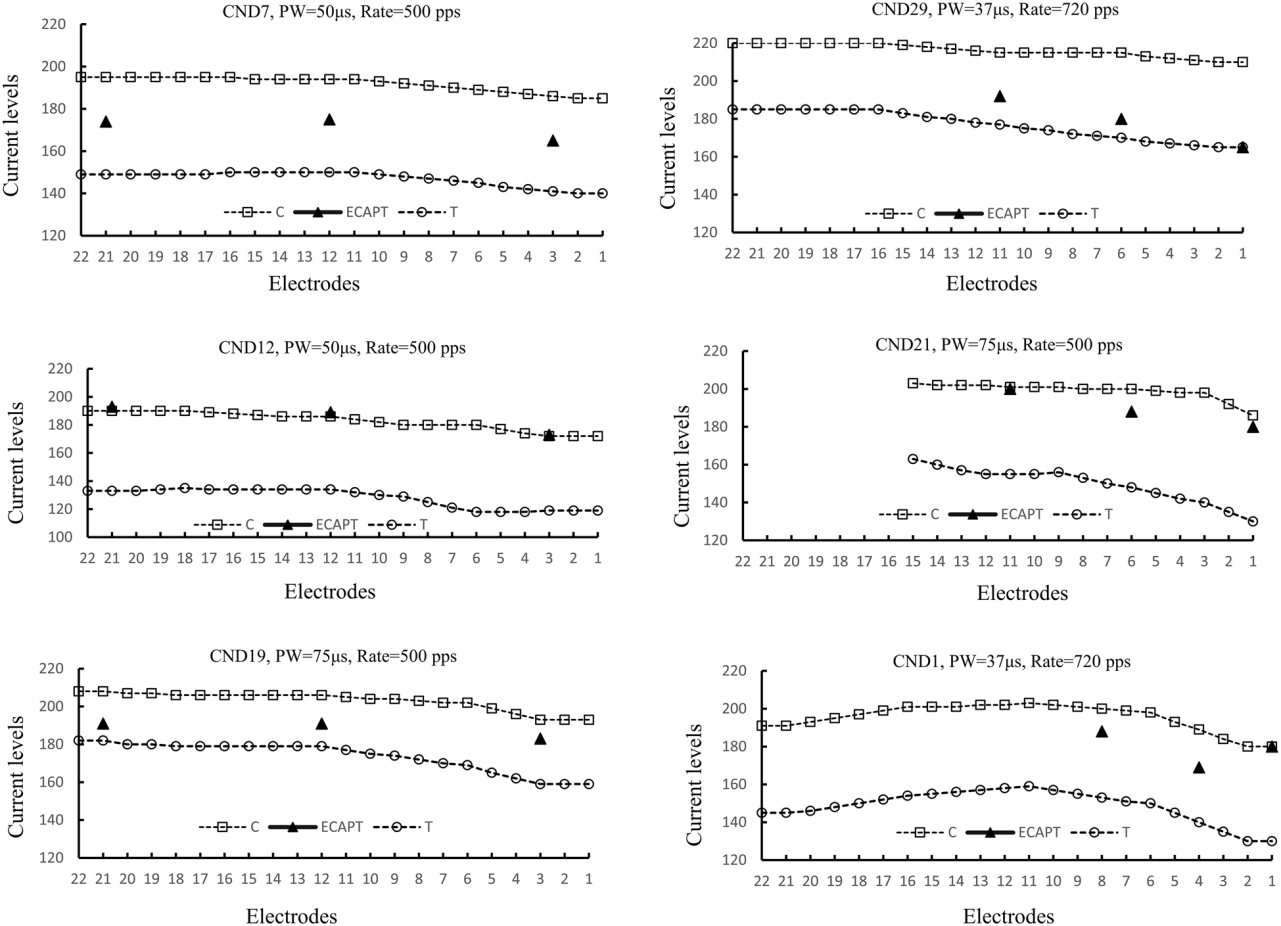
This figure displayed daily MAPs for six participants. The pulse widths and stimulation rates used in each speech processor are displayed at the top of each panel. For CND21 electrodes 16 to 22 were deactivated due to facial stimulation. For other children, all electrodes were activated in daily MAPs. The position of ECAP threshold to T- and C-levels of the MAPs were plotted. The square represents the C-levels (C); the triangle represents the ECAP thresholds (ECAP T); and the circle represents the T-levels (T).

Inter-participant and cross-electrode variability of the relationship between ECAP thresholds and behavioral T-levels was also observed. Figure 6 shows the current levels for ECAP thresholds and MAP’s T/C-levels on each tested electrode in six representative children with CND. The pulse widths and stimulation rates used in individual participants are displayed at the top of each panel. For most children, MAP's T/C levels decreased from electrode 22 to electrode 1. However, a few children had the highest MAP's T/C levels at the middle part of the electrode array. The left panel displays three children whose ECAPs could be recorded at all electrodes, and the right panel displays three children whose ECAPs could only be recorded at partial of the electrodes. For most children (e.g., CND7, CND29, CND12, and CND21), the ECAP threshold profiles followed their behavioral T-levels. However, the degree of ECAP thresholds for predicting T/C-levels varied among individual participants. For CND7, ECAP thresholds fell at the middle of the MAP’s dynamic range. Participant CND29 showed ECAP thresholds closely approximated to the T-levels, while CND12 and CND 21 had ECAP thresholds close to MAP C-levels. There were still a few participants whose ECAP thresholds profiles did not follow T or C levels in their MAPs (e.g., CND19 and CND1). For these two participants, ECAP thresholds fell at the dynamic range of MAPs but were irregular.

## Discussion

The primary aim of this study was to explore the correlation between the ECAP threshold and behavioral T-levels in children with CND implanted with Cochlear^®^ Nucleus devices. The results of this study revealed a significant correlation between ECAP thresholds and behavioral T-levels, which provides important information regarding the use of the ECAP threshold to assist programming a CI process in children with CND.

To date, whether ECAP responses could be used to assist programming the speech processor in patients with CND has not been reported. This is likely due to the difficulty in recording ECAP responses in these patients. Typically, it is challenging to record neural responses using the automatic neural response telemetry technique in children with CND^5,17^. Based on previous report^18^, using specific parameters can make the recording of ECAPs more successful. In addition, many children with CND cannot cooperate with the behavioral test even after a long period of CI use due to the limited benefits. In this study, behavioral and ECAP thresholds were tested 1 year or later after the initial activation of the CI. This was due to several reasons. First, it had been reported that the ECAP responses tend to change during the first few months after implantation^19,20^. Second, specific parameters were used in the MAP for individual patient. According to our experience, it would take about 6 months or longer to confirm whether these parameters were appropriate for the patients. Additionally, Buchman et al. reported that a stable MAP is usually achieved 3–6 months after initial stimulation^21^. Moreover, it was difficult for most patients with CND to cooperate with behavioral testing shortly after implantation.

Our results showed significant correlations between ECAP thresholds and behavioral T-levels at different electrode locations along the electrode array. This result was consistent to those of previous studies for children with normal-sized cochlear nerve^22^. Additionally, the mean ECAP thresholds fell at the upper panel of the dynamic range of participants’ daily MAPs. These results were similar to those tested in SNHL children with normal-sized cochlear nerves. Previous studies also reported that ECAP threshold often fell at the upper panel of the MAP dynamic range in children^11,13^. Furthermore, similar to previous studies^13^, inter-subject variability of ECAP threshold to behavioral T-level relationship had also been observed in this study. As it shown in Fig. 6, ECAP thresholds might closely approximate to the MAP’s T-levels, or to the MAP’s C-levels, or irregular fell at the dynamic range of the MAP. These indicates that although significant correlations were observed between ECAP thresholds and behavioral T-levels, ECAP thresholds could not accurately predict MAP’s T-levels for an individual child with CND.

However, the ECAP threshold could provide some meaningful indications for programming stimulation levels for children with CND. First, the ECAP thresholds were equal or higher than the behavioral T-levels in all tested electrodes. This suggests that the ECAP threshold might provide an indicator of the highest T-level, which means that T-levels are generally set below the ECAP thresholds in daily MAPs for children with CND. Furthermore, ECAP thresholds were at the upper panel in approximately 70% of the tested electrodes in patients with CND. These results are particularly important for children with CND who cannot provide any behavioral responses. ECAP thresholds should at least help provide an objective baseline to assist programming stimulation levels in patients with CND.

Additionally, ECAP thresholds could assist audiologists in selecting appropriate pulse widths for children with CND. Results of this study showed that larger charge levels were used in the MAPs for children with CND than for other SNHL children with normal-sized cochlear nerves^20,23^. Other studies also reported that children with CND required greater charge per unit phase^21,24^. This might be due to the small number of spiral ganglion neurons in the cochleae of children with CND. Typically, the pulse with would be increased in order to elevate the stimulation level in clinic programming. However, it was not that the larger the pulse with the better the outcomes. As previous study reported that increasing the pulse width did not improve the responsiveness of the cochlear nerve to electrical stimulation for children with CND^25^. Moreover, due to the electrical leakage occurring at the neural membranes, a shorter pulse width was more effective than a longer pulse width in stimulating the cochlear nerve^26^. Since ECAP thresholds were significant correlated with behavioral T-levels, an appropriate pulse width for the MAP could be selected based on the ECAP results. We recommend using the smallest pulse width that could provide enough stimulation charge.

In this study, both the MAP’s C- and T-levels tended to increase as the electrode locations moved from basal to apical relative to the cochlea. This characteristic is inconsistent to other congenital SNHL children. In Allam’s and Park’s studies, both MAPs’ T- and C-levels were higher at basal than those at apical electrode locations^15,27^. This unique feature in the MAPs of children with CND might be attributed to the characteristic of the damage of the cochlear nerves in these children. Previous studies shown that the damage to the cochlear nerve in children with CND tended to increase from basal to the apical direction of the cochlea^7,8^. Thus, for patients in whom ECAP responses could not be recorded from all electrodes, the ECAP thresholds recorded at the most apical electrodes could serve as a reference to set stimulation levels for electrodes without ECAP responses. Moreover, for most participants in this study, the contour of the ECAP thresholds was similar to that of the T-levels across the electrodes. Thus, for these children with CND, a rough outline of the MAP could be determined by ECAP thresholds and behavioral T-levels from three electrodes along the electrode array. This indicates that for children with CND who cannot provide sufficient behavioral responses, ECAP thresholds can help audiologists select stimulation levels more efficiently.

This study has some potential limitations. Firstly, since most of children with CND could not precisely express their experience of what constituted loud or comfortable sounds, we only focused on the correlation between ECAP thresholds and behavioral T-levels. Secondly, only three electrodes were tested for each participant due to limited patient compliance and inconsistent electrodes with ECAP response among the participants. Moreover, the stimulation rates used in MAPs varied among individual child which might influence the correlation between the ECAP thresholds and behavioral T-levels. Previous studies have shown that the stimulation rates may affect the correlation between ECAP thresholds and behavioral responses^28^. The relation between ECAP responses and responses at different stimulation rates needs to be further evaluated in studies with a larger participant base.

## Conclusion

ECAP thresholds were significantly correlated with behavioral responses of the auditory nerve in children with CND. For children with CND who cannot provide sufficient behavioral responses, ECAP responses may provide a useful baseline for selecting appropriate stimulation levels in the MAP.

## Data Availability

Anonymized data are available upon request from the corresponding author.
